# Functional outcomes of all-inside arthroscopic anterior talofibular ligament repair with loop suture versus free-edge suture

**DOI:** 10.1186/s13018-022-03402-z

**Published:** 2022-11-19

**Authors:** Shi-Ming Feng, Chang-Qing Shao, Qing-Qing Sun, Francesco Oliva, Nicola Maffulli

**Affiliations:** 1 Orthopaedic Department, Sports Medicine Department, Xuzhou Central Hospital, Xuzhou Clinical College of Xuzhou Medical University, No. 199, the Jiefang South Road, Xuzhou, 221009 Jiangsu People’s Republic of China; 2 Department of Musculoskeletal Disorders, Faculty of Medicine and Surgery, University of Salerno, Salerno, Italy; 3 Guy Hilton Research Centre, School of Pharmacy and Bioengineering, Keele University, Stoke-on-Trent, Staffordshire ST4 7QB England, UK; 4 Centre for Sports and Exercise Medicine, Barts and The London School of Medicine and Dentistry, Mile End Hospital, 275 Bancroft Road, London, E1 4DG England, UK

**Keywords:** Chronic lateral ankle instability, Arthroscopic anterior talofibular ligament repair, Loop suture, Free-edge suture

## Abstract

**Background:**

Anatomic repair of anterior talofibular ligament (ATFL) is used to manage chronic lateral ankle instability (CLAI). However, the optimal suture configuration used to repair the ATFL is not yet determined. It remains unclear whether suture configuration affects clinical outcomes in such patients.

**Purpose:**

To compare the functional outcomes of all-inside arthroscopic ATFL repair using either a loop suture and or a free-edge suture configuration in CLAI patients.

**Study Design:**

Cohort study; Level of evidence, 3.

**Methods:**

This retrospective cohort study included 71 patients with CLAI who had undergone an all-inside arthroscopic ATFL repair procedure with either loop suture (*n* = 36) or free-edge suture (*n* = 35) from February 2016 to July 2018. Comparable pre-operatively, the Visual analogy score (VAS), American Orthopedic Foot and Ankle Society scoring system (AOFAS), Karlsson Ankle Functional Score (KAFS) scoring system, Anterior Talar Translation (ATT) and Active Joint Position Sense (AJPS) were used to evaluate postoperative ankle function.

**Results:**

There were no postoperative wound complications, implant reactions, or neurological or vascular injuries. Postoperative hospitalization, VAS, AOFAS, KAFS, AJPS and the time of return to sport were similar between the loop suture group and free-edge suture group. Requiring a longer procedure time, patients with loop suture configuration achieved better ATT.

**Conclusion:**

All-inside arthroscopic ATFL repair procedure for CLAI treatment provides better ATT and comparable functional outcomes when a loop suture configuration is used instead of a free-edge suture configuration. A statistical difference in ATT was observed. Given the relatively short follow-up, it is questionable whether this will have any clinical relevance.

## Introduction

Ankle injuries are common in all sports, and inversion injury of the ankle is the most frequent ankle injury [1–5]. The anterior talofibular ligament (ATFL), calcaneofibular ligament and posterior talofibular ligament are the main ligament structures to maintain the stability of the lateral ankle joint. Injuries to the ATFL account for about 62% of the injuries to the lateral ankle ligament complex [6, 7]. After 3 to 6 months of appropriate conservative treatment and rehabilitation, most patients with ATFL injury can regain satisfactory functional results [8]. However, even after appropriate rehabilitation, residual lateral ankle pain, repeated ankle sprain and giving way are observed in 10% to 12% of the patients, who develop chronic lateral ankle instability (CLAI) [1, 9]. Surgical management is recommended for symptomatic CLAI [10, 11].

Anatomical repair of the ATFL is the mainstay of surgical strategies in CLAI management [12]. Satisfactory functional outcomes are obtained following open surgery, and, when the procedure is preceded by arthroscopic surgery, intra-articular lesions can be successfully managed [13, 14]. Recently, arthroscopic management of the ligamentous lesions of the ankle has become more popular [15–17]. The ankle joint is a major load-bearing joint of the lower limb, and compressive stresses during sport and exercise can reach 3 to 5 times the body weight [1]. Arthroscopic surgical procedures are however more technically challenging, and different suture configurations are in use. To date, loop suture and free-edge suture configurations are both used for arthroscopic ATFL repair [10, 18]. However, there are few publications addressing the issue of differences in functional results between the use of loop suture and free-edge suture.

The purpose of this study was to compare the clinical results in terms of ankle function, stability and proprioceptive recovery in 71 patients with CLAI treated by all-inside arthroscopic ATFL repair using loop suture or free-edge suture in our department from February 2016 to July 2018.

We wished to test the null hypothesis of no difference in outcome of the two arthroscopic suture configurations in patients with CLAI.

### Patients and methods

The institutional review boards of our hospital approved the study. The study was a retrospective cohort study evaluating the outcomes of patients undergoing arthroscopic anatomical repair of ATFL with either loop suture or free-edge suture configuration. All patients provided a signed informed consent as well as consents for the Health Insurance Portability and Accountability Act to participate in this study.

### Patient selection

**Fig. 1:**
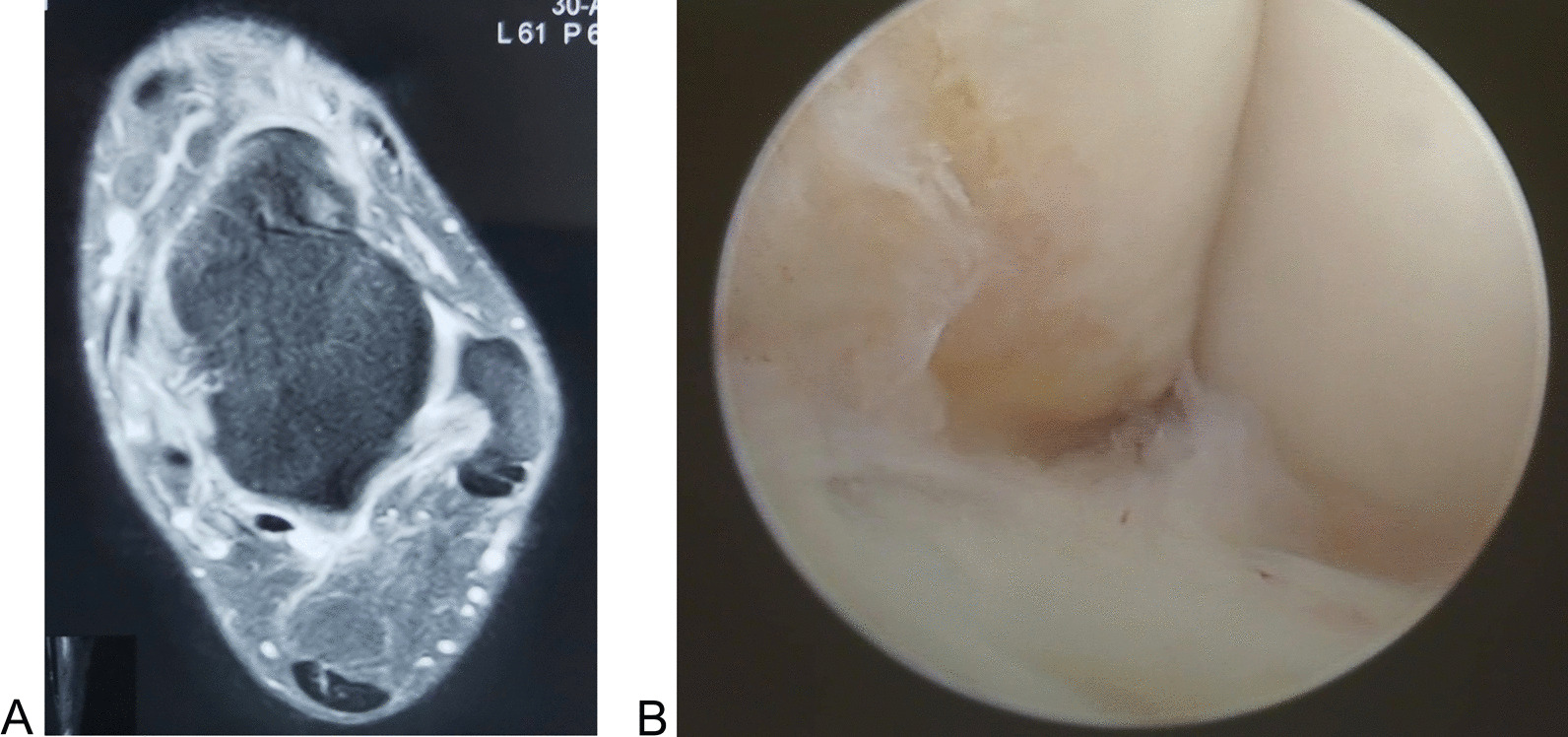
ATFL injury confirmed at the magnetic resonance imaging (**A**) and arthroscopy (**B**)

We included patients who satisfied the following criteria: (1) skeletally mature patients who were diagnosed with CLAI after failure of at least 6 months of conservative management; (2) patients with giving way, and ankle sprained more than twice in the previous 6 months; (3) positive pre-surgery anterior drawer test [19]; (4) pre-operative Magnetic Resonance Imaging show injury of the ATFL (Fig. 1A); (5) patients with unilateral ankle ATFL injury (non-avulsed fracture) but no calcaneofibular ligament injury (confirmed at arthroscopy) (Fig. 1B); (6) patients who had received all-inside arthroscopic ATFL repair procedure using the loop suture or free-edge suture configuration; (7) patients with complete surgical and follow-up data, followed up for at least 12 months.

We excluded patients who satisfied the following criteria: (1) patients with generalized ligamentous laxity; (2) patients who presented CLAI combined with foot and ankle deformity, abnormal hindfoot alignment, previous foot and ankle surgery, and other ligament injuries; (3) patients with osteochondral injury greater than 15 mm in diameter in any direction, or which required osteochondral transplantation, or in whom CLAI was associated with ankle osteoarthritis/ talar cyst; (4) patients with an os subfibulare; (5) patients with severe comorbidities who could not tolerate the surgery; (6) patients who suffered secondary injury of the ankle or who underwent revision surgery.

### Participants

**Fig. 2:**
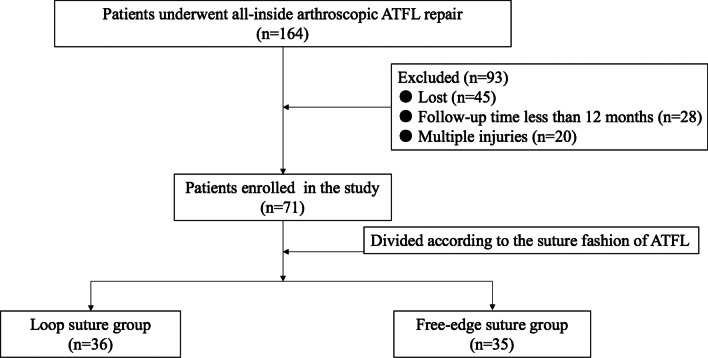
Flow diagram of the study

From February 2016 to July 2018, 164 consecutive CLAI patients underwent all-inside arthroscopic ATFL repair with loop suture configuration or free-edge suture configuration by a senior surgeon with extensive experience in foot and ankle surgery. A total of 93 patients did not meet the inclusion criteria and were excluded. Of these patients, 45 were lost during the postoperative follow-up period. The follow-up period was less than 12 months in 28 patients, and the ATFL injury was combined with other injuries in 20 patients. A total of 71 patients satisfied our inclusion criteria and were enrolled in the present study (Fig. 2).

After communication (suture fashion, operative time, currently reported outcomes) with the doctor, patients were invited to choose between the free-edge configuration or the loop configuration. The patients who were undecided tossed a coin and were allocated to undergo one or the other technique.

Patients were divided into two groups based on the ATFL suture configuration. In a total of 36 patients the ATFL was sutured using a loop configuration. In the remaining 35 patients, a free-edge configuration was used to suture the ATFL.

In the loop suture group, there were 22 male patients and 14 female patients, aged 19 to 52 years (average age, 33.83 years). The injury occurred during sports (*n* = 25) or following traffic accidents (*n* = 11). The right ankle was involved in 23 patients, and left ankle in 13 patients. The procedure was performed after a mean duration of 15.75 ± 3.16 months (range, 12–22 months) from the index injury. In the free-edge suture group, there were 20 male patients and 15 female patients, aged 18 to 54 years (average age, 35.71 years). The injury occurred during sports (*n* = 24) or following traffic accidents (*n* = 11). The right ankle was involved in 20 patients, and the left ankle in 15 patients. The procedure was performed after a mean of 15.63 ± 2.84 months (range, 12–22 months) from the index injury (Table 1).

**Table 1:** Characterization of the Sample

Variable	Loop suture group (*n* = 36)	Free-edge suture group (*n* = 35)	*P*^a^value
Age, yr	33.83 ± 8.59	35.71 ± 9.00	0.371^b^
Sex			0.116^c^
Male	22	20	
Female	14	15	
BMI, kg/m^2^	24.63 ± 2.45	24.26 ± 2.71	0.552^b^
Side			0.338^c^
Right	23	20	
Left	13	15	
VAS, mm	6.00 ± 1.94	6.17 ± 1.93	0.710^b^
AOFAS	70.47 ± 9.06	72.03 ± 9.30	0.477^b^
KAFS	67.03 ± 8.88	67.34 ± 11.23	0.896^b^
ATT	7.19 ± 1.37	7.20 ± 1.32	0.986^b^
Time between injury and surgery, mo	15.75 ± 3.16	15.63 ± 2.84	0.865^b^
Length of surgicalprocedure, min	47.61 ± 4.49	43.46 ± 5.70	0.001^b^
Hospital stay, days	3.58 ± 0.60	3.60 ± 0.65	0.911^b^
Follow-up, mo	22.97 ± 7.11	22.46 ± 7.22	0.763^b^

*BMI* body mass index; *VAS* visual analogue scale; *AOFAS* American orthopaedic foot and ankle society; *KAFS* Karlsson ankle function score; *ATT* anterior talar translation

^a^A value *p* < 0.05 was set as statistically significant

^b^t test

^c^Pearson χ^2^ test

### Surgical technique

With the patient supine on the operating table, general anesthesia and lumbar plexus-sciatic nerve block were performed. A 7 cm pillow was placed under the ipsilateral hip, and the affected ankle was placed over the distal edge of the operating table. A pneumatic tourniquet was placed at the root of the thigh and inflated to 300 mmHg after exsanguination.

**Fig. 3:**
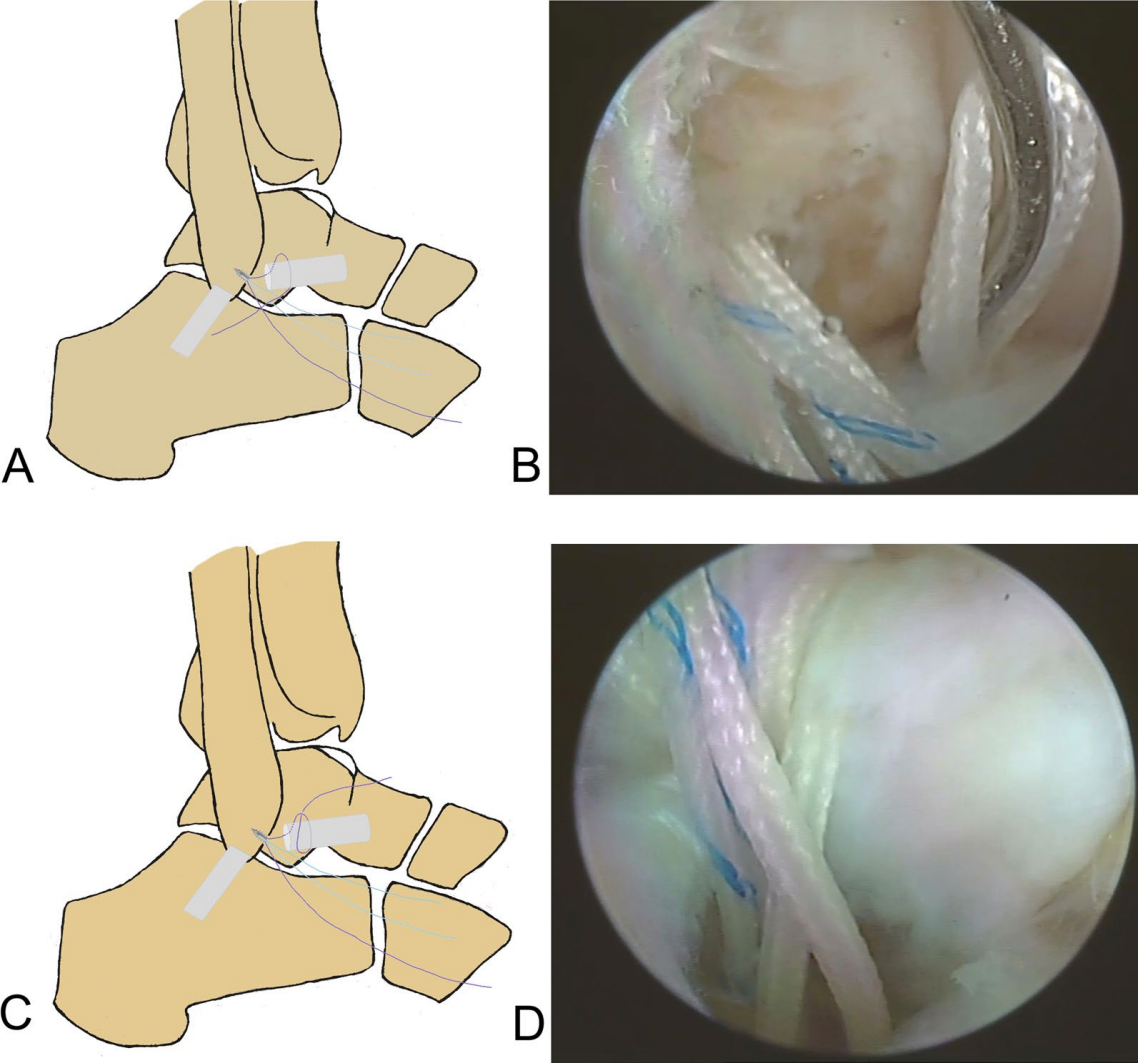
Surgical diagrams of all-inside arthroscopic ATFL repair with loop suture configuration (one suture arm sutured). **A** and **B**: one suture arm (purple) was passed through the distal ATFL remnant and a loop was created in the lateral gutter. **C** and **D**: the free end of the suture arm (purple) was passed through the created loop, and the ATFL would be self-cinching sutured when the free suture arm was pulled

**Fig. 4:**
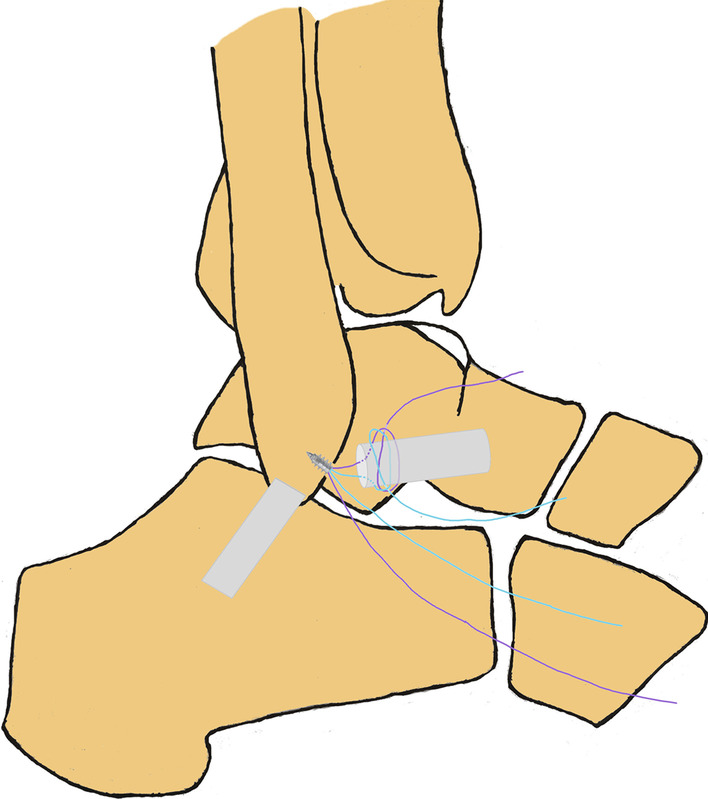
Surgical diagram of all-inside arthroscopic ATFL repair with loop suture configuration (two suture arms sutured)

**Fig. 5:**
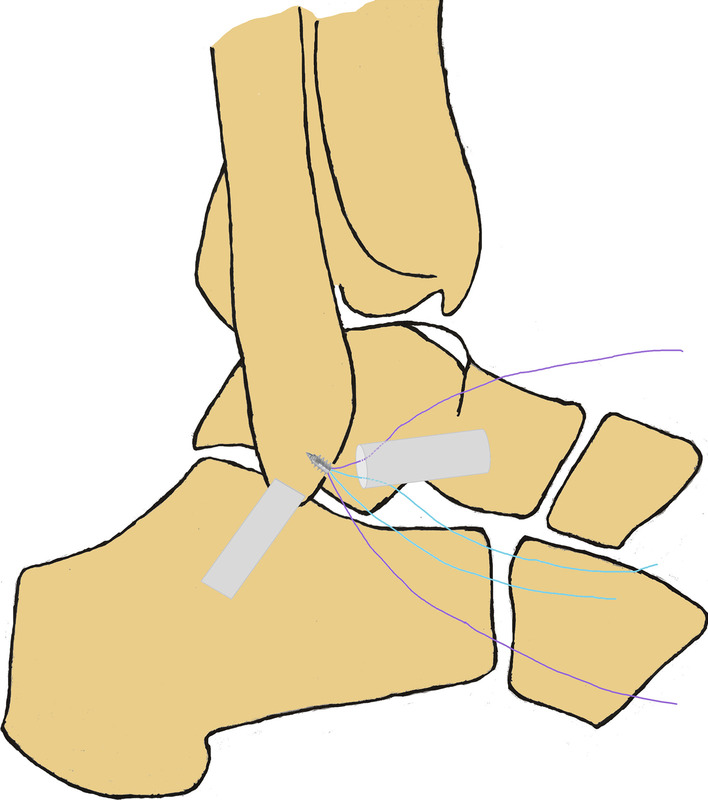
Surgical diagram of all-inside arthroscopic ATFL repair with free-edge suture configuration

A standard anteromedial viewing portal was established, and intra-articular lesions were evaluated arthroscopically. Under direct arthroscopic vision, an anterolateral portal was established lateral to the peroneus tertius. The intra-articular lesions were managed using a 3.5 mm shaver inserted through this portal, and the ATFL footprint on the fibula was exposed. The cartilage of the talus was evaluated by gentle probing. The ATFL was identified and its tension and quality were assessed. An anterior subtalar portal was produced if the ATFL was difficult to identify and assess. A bleeding bony surface at the footprint region of the fibula was produced using a motorized burr. To protect the remnant of the ATFL, a polydioxanone suture can be used to pull the ATFL. A double loaded suture anchor (Fastin RC 3.5 mm, Smith & Nephew, Andover, MA) was inserted into the mid-portion of the footprint through the anterior subtalar portal or an accessory anterolateral portal. The anchor was inserted at an angle of 30° to 45° from the longitudinal axis of the fibula. The proximal remnant of the ATFL was grasped with a grasper, and maintained under gentle tension. The ATFL was sutured using a loop (Fig. 3 and Fig. 4) or a free-edge configuration [10] (Fig. 5). Finally, after confirming under arthroscopic vision that the suture held, the suture limbs were sutured with a knot pusher with the ankle slightly dorsiflexed and everted. The anterior drawer test and the ankle varus stress test were performed under direct arthroscopic vision. After confirming that the sutures held, the arthroscope was withdrawn and the incision was closed with a 3–0 monofilament.

### Postoperative management

A total of three doses of antibiotics were administered within 24 h after the operation. A short leg brace was used to immobilize the ankle joint for 2 weeks in slight dorsiflexion and eversion. Patients performed functional exercises under the guidance of a physical therapist. On the second day after surgery, patients were advised to perform gentle non-weight-bearing mobilization isometric exercises of the lower limb muscles. Two weeks later, weight-bearing functional exercises were initiated, and an AircastTM (DJO, Vista, CA, USA) boot was used. Physical activities were encouraged after removing the Aircast boot 6 weeks later.

### Postoperative follow-up and observational indexes

All the patients were followed up as outpatients. All the postoperative indexes were recorded at each follow-up appointment after the index surgery. The surgical duration, hospital stay duration, wound healing and complications were recorded. The anterior drawer test was performed and compared with the uninjured limb. The visual analog score (VAS), American Orthopedic Foot and Ankle Society scoring system (AOFAS), Karlsson Ankle Functional Score (KAFS) scoring system and Anterior Talar Translation (ATT) were administered to assess ankle function and therapeutic efficacy. Ankle proprioception was assessed using the Active Joint Position Sense (AJPS) [20]. The realization of AJPS: with the patients seated on a height-adjustable table, the affected lower limb was placed at a 90° angle from the hip, knee, and ankle, respectively. At the assistance of the footplate, the operated ankle was passively placed in 10° and 20°of inversion and plantar flexion, respectively. Patients were then asked to actively place the foot in the above positions. Every position was tested three times, and the average value was observed. All the above outcomes were evaluated by the same experienced ankle surgeon who did not participate in the index surgery, and was blinded to the procedure performed.

### Statistical analysis

The SPSS 19.0 statistical software was used for analysis. Quantitative variables were expressed as means and standard deviations. Surgical duration, hospital stay duration, VAS, AOFAS, KAFS, ATT and AJPS scores in the two groups were compared and analyzed using the t test (symmetric distribution) or the Mann–Whitney test (asymmetric distribution). The Pearson chi-square test was used to compare the categorical variables. A post hoc power analysis was performed. A difference of *p* < 0.05 was deemed as statistically significant.

## Results

The surgical procedure lasted from 40 to 56 min, with a mean of 47.61 ± 4.49 min. The length of postoperative hospital stay ranged from 3 to 5 days, with a mean of 3.58 ± 0.60 days. The mean follow-up duration was 22.97 ± 7.11 months (range, 12–37 months).

The surgical procedure lasted from 36 to 59 min, with a mean of 43.46 ± 5.70 min. The length of postoperatively hospital stay ranged from 3 to 5 days, with a mean of 3.60 ± 0.65 days. The mean follow-up duration was 22.46 ± 7.22 months (range, 12–36 months) (Table 1).

Wounds healed by first intention in both groups, and no infections were observed. At the last follow-up appointment, no implant rejection or suture reaction was encountered. All anchors were fixed firmly without obvious signs of failure or evidence of detachment with the assessment of X-ray and ultrasound.

### Analyses

There was no significant difference between two groups regarding age, sex, BMI, side, length of time between injury and surgery, length of surgical procedure, hospital stay, and length of follow-up. The pre-VAS, AOFAS, KAFS and ATT were similar between the two groups. The loop group presented longer surgical duration compared with the free-edge suture group (Table 1).

At final follow-up, similar improvements of VAS, AOFAS, KAFS and ATT were recorded in both groups (Table 2). The mean ATT was 3.22 ± 0.83 mm (range, 2–5 mm) in the loop suture group and 3.60 ± 0.70 mm (range, 2–5 mm) in the free-edge suture group. The difference was statistically significant (*p* = 0.041) (Table 2). Group sample sizes of 36 and 35 achieve less than 20.00% power (AOFAS, KAFS, and AJPS, respectively) to reject the null hypothesis of equal means, with a significance level (alpha) of 0.050 using a two-sided two-sample unequal-variance t-test.

**Table 2:** Functional outcomes comparison of the two groups

Variable	Loop suture group (n = 36)	Free-edge suture group (n = 35)	*P*^a^value	Power^d^
VAS, mm	0.00 (0.00–1.00)	0.00 (0.00–1.00)	0.896^c^	
AOFAS	91.89 ± 4.75	91.94 ± 3.83	0.958^b^	0.057
KAFS	90.58 ± 4.56	90.29 ± 4.19	0.776^b^	0.059
ATT, mm	3.22 ± 0.83	3.60 ± 0.70	0.041^b^	
*AJPS, degree*				
Inversion 10°	7.44 ± 1.30	7.51 ± 1.12	0.809^b^	0.057
Inversion 20°	17.67 ± 1.69	18.06 ± 1.51	0.309^b^	0.173
Plantar flexion 10°	7.69 ± 1.12	7.69 ± 1.18	0.975^b^	0.050
Plantar flexion 20°	18.50 ± 1.21	18.26 ± 1.40	0.436^b^	0.119

*VAS* visual analogue scale; *AOFAS* American orthopaedic foot and ankle society; *KAFS* Karlsson ankle function score; *ATT* anterior talar translation; *AJPS* active joint position sense

^a^A value *p* < 0.05 was set as statistically significant

^b^t test

^c^Mann–Whitney test

^d^Power is computed to reject the null hypothesis of equal means

Considering return to sports, 24 patients resumed their pre-injury sports activities and 12 patients returned to non-intense activities because of fear of secondary injury in the loop suture group. In the free-edge suture group, 22 patients returned to their pre-injury sports and 13 patients returned to non-intense activities, with no statistical difference between the two groups. The mean time of the patients return to sports activities was 9.53 ± 1.36 week (range, 8–12 week) in the loop suture group and 9.31 ± 1.28 mm (range, 8–12 week) in the free-edge suture group. No significant difference was observed (*p* = 0.498).

## Discussion

The key finding of the current study was that all-inside arthroscopic ATFL repair surgery for CLAI with a loop suture configuration provides similar functional results compared with free-edge suture. The postoperative hospitalization, VAS, AOFAS, KAFS, AJPS and the time of return to sport were all similar between the loop suture group and free-edge suture group at a mean follow-up of 22 months. Patients with loop suture of the ATFL achieved statistically better ATT compared with free-edge suture (3.22 mm vs. 3.60 mm, difference 0.38 mm). Given the relatively short follow-up, it is hard to know whether this will have any clinical relevance.

The ATFL is the most commonly injured ligament in ankle sprains. Conservative treatment, involving a brief period of immobilization followed by 3 to 6 months targeted rehabilitation, can achieve good functional recovery, but up to 30% of patients may develop CLAI. Surgical management is recommended after failure of 6 months of conservative management in CLAI patients [10, 21–24]. Broström first reported ATFL repair for CLAI treatment, and in the next decades, ATFL repair is proved as the optimal reliable option [25, 26]. The ATFL remnant is of good quality in most CLAI patients, and direct anatomical repair is generally possible [27], with comparable stability and functional outcomes following arthroscopic ATFL and open repair [28, 29].

Isolated anatomic repair of the ATFL produced excellent long-term functional outcomes [19]. However, the optimal suture configuration to repair the ATFL has not been established. Various suture techniques have been reported to repair the ATFL for patients with CLAI. Zhou et al. [30] reported ATFL repair with free-edge suture configuration using one anchor in 20 consecutive patients: the AOFAS score increased from 60.82 to 90.18 at a mean follow-up of 30.27 months. Li and coworkers [31] treated 20 CLAI patients with all-in arthroscopic ATFL repair surgery and single anchor fixation. The ATFL was sutured in free-edge suture and horizontal mattress suture fashion. At final follow-up, the AOFAS score increased to 90 and KAFS increased to 80. Martin et al. [32] evaluated the functional outcomes of 93 patients treated with arthroscopic ATFL repair (sutured with free-edge fashion) and augmentation. The foot and ankle disability index improved from 67 preoperatively to 87 and 90 postoperatively at the follow-up point of 6 and 12 weeks, respectively. Lee et al. [25] used a knotless anchor loaded with 2 strands in all-inside arthroscopic ATFL repair. The ATFL was sutured using a loop suture configuration. The AOFAS score increased from 62.5 to 86.7 and the KAFS score from 43.5 to 76.5.

Comparable results could be obtained after arthroscopic ATFL repair with or without augmentation. Qin et al. [18] performed an all-inside intra-articular lasso-loop stitch technique for ATFL repair in 39 patients, who were followed up for a mean of 28.23 months. The KAFS score improved to 90.26, and lasso-loop stitch technique proved to be a reliable procedure for ATFL repair.

The present study demonstrated that the all-inside arthroscopic ATFL repair improved functional results of AOFAS, KAFS, ATT and AJPS regardless of the configuration used (loop suture or free-edge). There was no significant difference in VAS, AOFAS, KAFS and AJPS, but the ATT in the loop suture group was significantly lower than in the free-edge suture group. To our knowledge, there is no definite answer regarding which suture fashion to select, and no previous study comparing arthroscopic ATFL repair with loop suture and free-edge suture has been performed. The AOFAS, KAFS, ATT and AJPS were evaluated to assess ankle function and proprioceptive recovery. The AOFAS, though widely used [33], is not validated, but it allows to compare different studies [34]. The KAFS is an 8-item questionnaire that evaluates pain, swelling, instability, stiffness, stair climbing, running, work activities, and use of ankle supports. As a patient self-reported questionnaire, KAFS was developed to evaluate the postoperatively ankle function and detect CLAI. KAFS is valid and reliable in the assessment of ankle function compared to AOFAS [11]. The ATT is the most commonly performed imaging measurement to assess ankle laxity stability using the Telos device (150 N force). In the present study, the ATT measurement was repeated twice by two surgeons, and the average values were recorded. Although there is a statistical improvement in the ATT, and the loop suture configuration produced significantly lower ATT than the free-edge suture configuration, the actual result is unlikely to have any clinical relevance [35]. As a commonly used index for ankle proprioceptive function evaluation, the AJPS was employed to assess the ankle proprioceptive function in our study. Additionally, the rate of return to sports is an indicator of the benefit of surgery [36–38]. Given the results of the present study, all-inside arthroscopic ATFL repair surgery for CLAI with loop suture and free-edge suture provided comparable ankle function.

This study has several strengths: (1) the postoperative ankle function, stability and proprioception were carefully assessed by fully trained surgeons; (2) the study was adequately powered, and a representative sample of patients was recruited; (3) all the procedures were performed by a fully trained orthopedic surgeon who is well beyond his learning curve and well versed in both suture configurations; (4) the rehabilitation protocol was homogeneous, and did not differ between the two groups of patients. We are however aware of the limitations of the present study: (1) AOFAS mainly focuses on the evaluation of pain, with less emphasis on the assessment of ankle stability. (2) the lack of biomechanical characterization of the two suture fashions represents a most important limitation; (3) furthermore, the limited number of functional outcomes analyzed might not comprehensively assess all aspects of ankle function; (4) being a retrospective study, inadvertent biases in data collection may have been introduced; (5) the mean follow-up time is 22 months after the surgery, and the clinical outcomes may vary with the time and the number of participants.

Nevertheless, within the limits outlined above, the results of the present investigation suggest that the suture configurations employed are both functionally effective, and the slight difference in mechanical results, though statistically significant, is likely not of any clinical relevance.

## Conclusion

Compared with the all-inside arthroscopic ATFL repair in free-edge suture configuration, the all-inside arthroscopic ATFL repair in loop suture configuration offers no short-term functional advantages in postoperative clinical outcomes, such as AOFAS, KAFS, AJPS and rate of return to sports. Patients with loop suture of the ATFL achieved statistically better ATT compared with free-edge suture (3.22 mm vs. 3.60 mm, difference 0.38 mm), but the difference is unlikely to be of any clinical relevance. Both all-inside arthroscopic ATFL repair using a free-edge or loop suture configuration are reliable procedures for the treatment of CLAI with satisfactory ankle function.

## Data Availability

Data are available from the co-corresponding authors following reasonable requests.
